# Frailty and Sarcopenia as a Geriatric Syndrome in Community-Dwelling Older Adults

**DOI:** 10.3390/ijerph16204013

**Published:** 2019-10-19

**Authors:** Hyuma Makizako

**Affiliations:** Department of Physical Therapy, School of Health Sciences, Faculty of Medicine, Kagoshima University, Kagoshima 890-8544, Japan; makizako@health.nop.kagoshima-u.ac.jp

Geriatric syndrome refers to a series of symptoms and observations caused by a variety of factors associated with aging, where the older adults show the treatment consciously or otherwise, and long-term care becomes important at the same time. With respect to geriatric syndrome, often the viewpoint involves the purpose of sustaining the capability for activities of daily living (ADL) and improving the quality of life (QOL), rather than just considering the disease condition according to the diagnostic name.

Notably, there are geriatric syndrome symptoms that are more frequent and are largely affected by long-term care, such as, disorders associated with mobility (remain secluded, or bedridden), falls, incontinence, and cognitive impairment. These are considered issues for which early measures should be incorporated into geriatric treatment. In recent years, measures against frailty and sarcopenia, which elevate the risk of increasing the need for long-term care, are being considered important.

Frailty progresses as vulnerability to stress increases due to declining physiological reserves with aging, making one prone to unhealthy conditions [1]. It is a factor that increases the risk of falling, disabilities in activities of daily living, onset of the need for long-term care, and death. Frailty as a concept includes not only physical problems, such as the representative decline in muscle strength, but also mental and physical problems, such as cognitive impairment and depression. It also includes social problems, such as solitary living and economic distress. Accordingly, it needs to be considered comprehensively. Frailty is said to be reversible. Therefore, it is expected that appropriate intervention for frail older adults will lead to improvements in physical function and daily living capabilities, help reverse frailty, and prevent the onset of functional disorders. For prevention and improvement of frailty, the key points will be active promotion of early identification of risk and desirable interventions as early measures.

“Sarcopenia” is a coined word, where “sarx (sarco)” means muscle and “penia” means “loss and reduction” in Greek, and it was first proposed by Rosenberg in 1989 [2]. Originally, it had a strong physiological nuance of decrease (atrophy) in muscle mass with aging. However, in addition to primary (aging) sarcopenia, a broader definition is taking hold, which includes as secondary sarcopenia the reduction in muscle mass and muscle strength and further muscle function loss, due to factors associated with activity, disease, and nutrition. In 2016, sarcopenia was registered in the International Classification of Diseases (ICD-10). It is now considered as a disease, and measures for the prevention, treatment, and improvement of sarcopenia are urgently needed in the future.

It has been reported that in frail older adults, exercise intervention is effective in improving physical functions, including gait speed [3]. However, the effects of the recommended specific event, frequency, and time have not been clarified. In addition to strength training, multifaceted exercise programs that incorporate flexible exercise, balance exercise, and aerobic exercise have also been reported to improve muscle strength in frail older adults. It has been suggested that interventions focused on the physical elements behind frailty (weight loss, muscle weakness, exhaustion, slowness, low physical activity) are effective, and comprehensive interventions incorporating as many of the elements as possible are desirable [4]. 

Resistance training and nutritional (protein) interventions are often selected as measures for prevention and improvement of sarcopenia. In a report on increased muscle mass and muscle strength in older adults, a high load of 70–80% of 1 repetition maximum (RM) was used. However, for older adults without exercise habits, it was reported that even with a load of 40–50% of 1 RM improvement in muscle function can be expected [5]. When setting a low load cannot be avoided, long-term and high frequency interventions may be necessary. Moreover, nutritional interventions for the purpose of promoting muscle protein synthesis (such as amino acid intake) are effective in increasing muscle mass and improving muscle strength. Accordingly, not only exercise interventions but also nutritional interventions undertaken concurrently are expected to be effective against sarcopenia.
